# Angiographic findings before and after the onset of brolucizumab-associated retinal vascular occlusion and intraocular inflammation

**DOI:** 10.1016/j.ajoc.2022.101521

**Published:** 2022-04-14

**Authors:** Sentaro Kusuhara, Kyung Woo Kim, Akiko Miki, Makoto Nakamura

**Affiliations:** Division of Ophthalmology, Department of Surgery, Kobe University Graduate School of Medicine, Kobe, Japan

**Keywords:** Brolucizumab, Retinal vascular occlusion, Intraocular inflammation, Fluorescein angiography, Indocyanine green angiography

## Abstract

**Purpose:**

To describe angiographic features of a case of delayed-onset retinal vascular occlusion and intraocular inflammation (IOI) following brolucizumab intravitreal injection (IVI).

**Observations:**

A 75-year-old woman with advanced age-related macular degeneration (AMD) complained of persistent distorted vision despite 1 aflibercept 2-mg IVI and subsequent 1 brolucizumab 6-mg IVI. At 20 days after brolucizumab IVI, clinical examination showed no signs of IOI, her right best-corrected visual acuity (BCVA) was 1.0 (Snellen equivalent, 20/20). Simultaneous fluorescein and indocyanine green angiography (FA/IA) performed 2 days later showed no abnormalities, but she noticed floaters and decreased vision in her right eye 5–6 hours after FA/IA. At 44 days after brolucizumab IVI, her right BCVA was 0.6 (Snellen equivalent, 20/33), and clinical examination revealed mutton-fat keratic precipitates, anterior chamber cells (2+), vitreous cells, vitreous haze (1+), and sheathed retinal vessels. FA showed filling defect and vascular staining/leakage at several retinal arteries and dye leakage from optic disc edge, where IA demonstrated dye staining as well. However, there was a retinal artery occlusion site which lacks angiographic signs indicative of active retinal vasculitis. The patient was diagnosed with retinal vascular occlusion and IOI which occurred approximately 3 weeks after brolucizumab IVI.

**Conclusions and importance:**

Delayed brolucizumab-associated retinal vascular occlusion and IOI can develop from a condition in which no apparent abnormal findings exist on FA/IA. Together with the fact that angiographic signs observed in this case were not severe enough to induce retinal artery occlusion, potent and prolonged vascular endothelial growth factor inhibition by brolucizumab IVI might have caused severe damage to retinal vascular endothelial cells. Then, the damage subsequently led to retinal vascular occlusions and enhanced immune reaction to brolucizumab. The latter would be enhanced through the migration of immune cells towards vitreous cavity being allowed by disrupted inner blood retinal barrier.

## 1 Introduction

With its approval by Food and Drug Administration (FDA) on October 7, 2019, the use of brolucizumab, a humanized single-chain variable fragment (scFv) that potently inhibits vascular endothelial growth factor (VEGF)-A, has been spreading worldwide for the treatment of advanced age-related macular degeneration (AMD).^1^ The efficacy of this newest anti-VEGF drug was supported by the data of phase 3 clinical trials, HAWK and HARRIER: (1) brolucizumab 6-mg intravitreal injection (IVI) (dosed every 8 or 12 weeks) showed non-inferiority to aflibercept 2-mg IVI (dosed every 8 weeks) in best-corrected visual acuity (BCVA) gain, and (2) brolucizumab IVI demonstrated greater reduction in retinal thickness compared to aflibercept IVI.^2^ Despite potentially highly rated efficacy of brolucizumab, concerns regarding the safety of brolucizumab IVI arose and have been prudently investigated by a safety review committee established by Novartis and other organizations. Among a wide variety of adverse events after brolucizumab IVI, intraocular inflammation (IOI), retinal vasculitis, and retinal vascular occlusion are getting attention as they are not frequently observed in other approved anti-VEGF drugs and may cause irreversible vision loss.^3^

A recent post hoc review of the phase 3 HAWK and HARRIER studies reported that the rate of the development of overall IOI, IOI + vasculitis, and IOI + vasculitis + occlusion was 4.6%, 3.3%, and 2.1%, respectively, and <1% of cases experienced visual acuity loss (≥15 ETDRS letters).^4^ In the analysis of the characteristics of 26 post-approval cases of retinal vasculitis after brolucizumab IVI performed by the American Society of Retina Specialists Research and Safety in Therapeutics Committee, 83% showed occlusive disease and 46% had a final BCVA of 20/200 or worse.^5^ Although the mechanisms for brolucizumab-associated IOI remain elusive, the most common and widespread plausible explanation for this delayed-onset inflammation would be type Ⅲ and/or Ⅳ hypersensitivities to brolucizumab^3^^,^^6^^,^^7^ as brolucizumab is thought to be more immunogenic than the other approved anti-VEGF drugs.^7^ However, without definite evidence that immune reactions account for all the abnormal ocular findings occurred after brolucizumab IVI, other mechanisms would be worth being considered.

We here describe a case of retinal vascular occlusion and IOI following brolucizumab IVI in which fluorescein angiography was performed before and after the onset of the disease. Angiographic features obtained from the case would surely contribute to deciphering the underlying mechanisms of this potentially vision-threatening ocular adverse event.

## 2 Case report

**Fig. 1:**
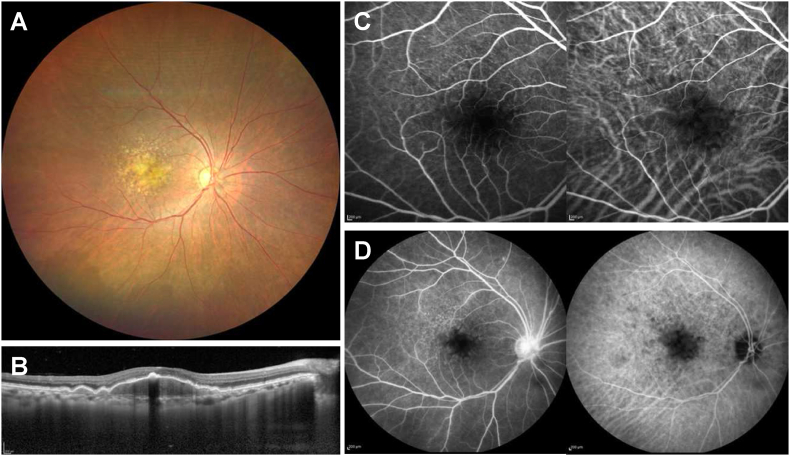
(A) Color fundus photograph of the right eye at 20 days after brolucizumab intravitreal injection (IVI). Intermediate and large drusen were present at the macula. (B) Optical coherence tomography at the same day revealed pigment epithelial detachments without concomitant subretinal fluid. (C) Early phase (33 secs) fundus angiographic images at 22 days after brolucizumab IVI. Fluorescein angiography (FA) (left) showed blocked hypofluorescence due to drusen and maintained retinal vessel perfusion at the macula. Indocyanine green angiography (IA) (right) demonstrated no remarkable findings except for hypofluorescence attributed to drusen. (D) Late phase (12 minutes) fundus angiographic images at 22 days after brolucizumab IVI. FA (left) showed no apparent abnormal findings other than hyperfluorescence at the optic disc. IA (right) demonstrated hypofluorescence corresponding to drusen.

A 75-year-old woman with bilateral advanced AMD complained of distorted vision in her right eye. A local doctor considered her symptom as being attributed to serous detachment over pigment epithelial detachment (PED). She received an aflibercept 2-mg IVI and subsequently brolucizumab 6-mg IVI 2 months later. Although the serous detachment disappeared, her visual symptom unchanged. Therefore, she was referred to our hospital (Kobe University Hospital) for further examination and treatment. She was a current smoker and had a medical history for systemic hypertension, dyslipidemia, and asthma. Her ocular history was unremarkable except for uneventful cataract surgery in both eyes, her family history was unremarkable, and she had no known food and drug allergies. She had taken budesonide formoterol fumarate hydrate, theophylline, l-carbocisteine, pravastatin sodium, azelnidipine, and candesartan cilexetil. On the first examination in our hospital, at 20 days after brolucizumab IVI, her best-corrected visual acuity (BCVA) was 1.0 (Snellen equivalent, 20/20) in the right eye and 1.2 (Snellen equivalent, 20/17) in the left eye. The intraocular pressure was 17 mmHg in both eyes. Slit-lamp examination showed no inflammatory signs in the anterior segment or vitreous cavity in both eyes. Dilated fundus examination showed no remarkable finding except for drusen and PEDs at the posterior pole in both eyes. Optical coherence tomography (OCT) B-scan images also visualized PEDs, but serous detachment was invisible in both eyes. Simultaneous fluorescein and indocyanine green angiography (FA/IA) preformed 2 days later also showed no apparent findings suggestive of IOI or active AMD (Fig. 1). Based on these findings, the patient was considered to have achieved inactive disease and was followed up without additional treatment.

**Fig. 2:**
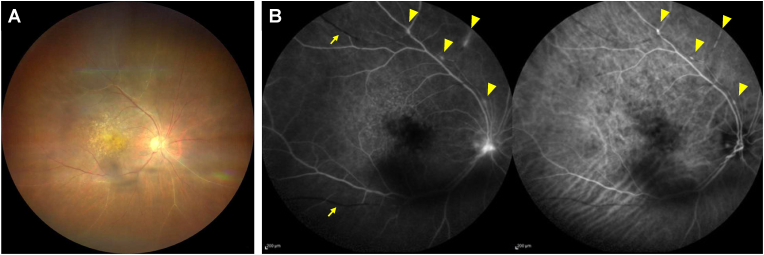
Fundus images of the right eye obtained 44 days after brolucizumab intravitreal injection. (A) Color fundus photograph showing vitreous opacities and sheathed retinal vessels. (B) Late phase (6 minutes) fluorescein/indocyanine green angiography (FA/IA) images. FA (left) showed filling defects (arrows), vascular staining/leakage (arrowheads) at retinal arteries, and dye leakage from optic disc edge. Please note that there is a filling defect site which lacks angiographic inflammatory signs requisite for retinal vascular occlusion secondary to retinal vasculitis. IA (right) demonstrated hyperfluorescent dye staining at the same sites as FA (arrowheads).

However, she noticed floaters and decreased vision in her right eye at home 5–6 hours after FA/IA. Because our hospital is far from her home, she visited the local doctor several days later and was diagnosed with having severe IOI in her right eye. Her right BCVA was 0.05 (Snellen equivalent, 20/400) at that time, and she was advised to administer 0.1% betamethasone sodium phosphate eye drop (6 times/day) and take kallidinogenase (150mg/day). At her next visit to our hospital (44 days after brolucizumab IVI), her right BCVA recovered to 0.6 (Snellen equivalent, 20/33), and clinical examination revealed mutton-fat keratic precipitates, anterior chamber cells (2+), vitreous cells, vitreous haze (1+), and sheathed retinal vessels. FA showed filling defect and vascular staining/leakage at several retinal arteries and dye leakage from optic disc edge. IA demonstrated dye staining at the same sites as FA. However, there was a filling defect site in which no angiographic signs were found indicating active retinal vasculitis responsible for retinal artery occlusion (Fig. 2). The left eye showed no inflammatory signs on clinical examination or FA/IA. The patient was diagnosed with retinal vascular occlusion and IOI which occurred approximately 3 weeks after brolucizumab IVI.

**Fig. 3:**
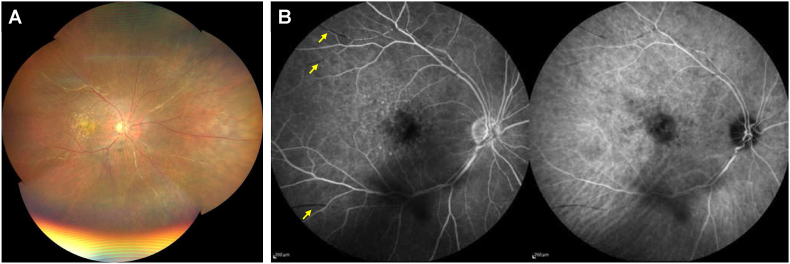
Color fundus photograph and fluorescein/indocyanine green angiography (FA/IA) images of the right eye acquired 79 days after brolucizumab intravitreal injection. (A) Montage color fundus image showing drusen and sheathed retinal arteries. (B) Late phase (11 minutes) FA/IA images. FA (left) demonstrated filling defects (arrows) without vascular leakage or staining. IA (right) showed no dye staining on retinal vessels.

**Fig. 4:**
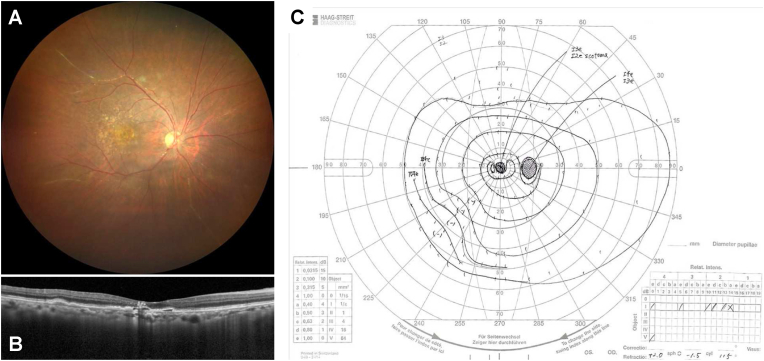
The results of ocular examinations in the right eye performed 135 days after brolucizumab intravitreal injection. (A) Color fundus photograph showing drusen and remaining sheathed vessels. (B) Optical coherence tomography demonstrating a flattened pigment epithelial detachment. (C) Goldmann perimetry showing preserved peripheral visual field.

To treat the IOI, oral prednisone 20mg/day (0.5 mg/kg/day) and aspirin (100mg/day) were started. Two weeks later, her symptom unchanged and her right BCVA remained 0.6 (Snellen equivalent, 20/33), but IOI was slightly improved: keratic precipitates were inconspicuous, anterior chamber cells decreased to 1+, and vitreous haze became faint. The dose of prednisone was reduced to 15 mg/day for the next 3 weeks, and sulfamethoxazole/trimethoprim and famotidine were added for the prophylaxis of side effects of corticosteroids. Three weeks later (at 79 days after brolucizumab IVI), her right BCVA was still 0.6 (Snellen equivalent, 20/33), but her symptom got better. Clinical examination showed no keratic precipitates, no anterior chamber cells, vitreous cells, faint vitreous haze, and sheathed retinal vessels. FA demonstrated unchanged filling defects but no vascular staining or leakage was observed, and IA showed no dye staining on retinal vessels (Fig. 3). The dose of prednisone was reduced to 5 mg/day for the next 3 weeks and was then stopped. Kallidinogenase, sulfamethoxazole/trimethoprim, and famotidine were also terminated at the same time, and the frequency of 0.1% betamethasone eye drop was decreased to 3 times daily. Although ocular slit-lamp and funduscopic findings remained unchanged, her right BCVA recovered to 0.8 (Snellen equivalent, 20/25). 5 weeks later (at 135 days after brolucizumab IVI). B-scan OCT showed a flattened PED probably due to brolucizumab therapy, and Goldmann perimetry disclosed preserved peripheral visual field (Fig. 4). Systemic workup to explore the cause of uveitis was performed which consists of hematologic and biochemical tests (including antinuclear antibodies, angiotensin converting enzyme, interferon gamma release assay, and serologic tests for syphilis), urinary test, chest X-ray, and electro-cardiogram test. However, it did not suggest any underlying systemic disease which could cause vascular occlusion or IOI.

## 3 Discussion

Currently, IOI and retinal occlusive vasculitis after brolucizumab IVI is one of the hot topics among retina specialists.^3^, ^4^, ^5^^,^^8^, ^9^, ^10^ The data from recently published case reports surely contribute to disclosing the underlying mechanisms of these potentially vision-threatening events.^3^^,^^5^^,^^6^^,^^11^, ^12^, ^13^, ^14^, ^15^, ^16^, ^17^, ^18^, ^19^ However, the lack of detailed clinical information just before the onset of IOI has been an obstacle to clarify the pathogenesis of brolucizumab-associated IOI. In our case, we luckily obtained angiographic features before and after the onset of retinal vascular occlusion and IOI following brolucizumab IVI.

In a retrospective case series from 10 United States centers, the mean interval from last brolucizumab IVI to diagnosis of retinal vasculitis and IOI was 30 days (range, 7–56 days).^3^ To the best of our knowledge, 12 papers have demonstrated ocular images on brolucizumab-related IOI and/or retinal vasculitis/occlusion so far,^3^^,^^5^^,^^6^^,^^11^, ^12^, ^13^, ^14^, ^15^, ^16^, ^17^, ^18^, ^19^ but no images were provided on FA/IA before its onset. There would be roughly two scenarios which can explain subsequent subclinical events occurred from brolucizumab IVI to the clinical diagnosis of IOI. One scenario is that subclinical ocular inflammation was induced as a result of type Ⅲ and/or Ⅳ immune reaction against the drug. As brolucizumab shows a higher molar dosing and longer half-life in the vitreous cavity than aflibercept,^1^^,^^20^ immune response to brolucizumab lasts long and damage to ocular tissues accumulates. Then, clinically detectable IOI and/or retinal vasculitis show up when the damage exceeds a threshold. In this scenario, some angiographic findings could be observed as in the remission phase of Behçet's uveoretinitis even if no inflammation was detected by slit-lamp biomicroscopy or funduscopy. The other scenario is related to potent and long-lasting VEGF inhibition following brolucizumab IVI. It is well known that VEGF is essential for the maintenance of normal vascular endothelial cell integrity.^21^ Hence, excessive and continuous VEGF inhibition caused by brolucizumab gradually leads to dysfunction of vascular endothelial cells, which results in thrombosis, vascular leakage, migration of immune cells towards vitreous body, etc. In this scenario, the key is whether retinal vascular endothelial cells retain their normal function, and therefore several events may simultaneously take place when damage to retinal vascular endothelial cells surpass a threshold. Because our case experienced retinal vascular occlusion and IOI approximately 3 weeks after brolucizumab IVI and FA/IA performed 5–6 hours before the onset did not show any apparent abnormal finding, the latter scenario could better explain the underlying events in this case.

Most of previous reports on brolucizumab-associated IOI and retinal occlusive vasculitis suggest that retinal vascular occlusion occurs secondary to retinal vasculitis. However, detailed observation of FA/IA images in our case made us aware that the idea is not always true. As shown in Fig. 2, some lesions lack angiographic findings of severe inflammation which is indispensable to cause retinal artery occlusion, and previous reports presented similar cases without discussing it.^3^^,^^17^ Some might raise a question; does retinal artery occlusion occur without retinal vasculitis? The answer is yes. Retinal specialists would have ever encountered such cases (e.g., ocular ischemic syndrome), and we previously reported a case of severely impaired retinal circulation during the systemic treatment of axitinib, a potent inhibitor of VEGF receptors 1, 2, 3.^22^ It is well known that VEGF inhibition causes disturbance of VEGF-dependent physiological functions of vessels and subsequent occlusion.^21^^,^^23^ From this viewpoint and the second scenario described above, we speculate that brolucizumab-associated IOI results from strong and long-lasting VEGF inhibition and occurs predominately in eyes with relatively impaired retinal vessels caused by various factors such as smoking, hypertension, and anti-cancer drugs. In fact, the current case was a current smoker and had been suffering from systemic hypertension and dyslipidemia. If our speculation holds true, an overdose use of the other anti-VGEF agents may cause IOI. In 2006, Rosenfeld et al. reported the results of a randomized clinical study of 3 different dose-escalating regimens of ranibizumab.^24^ In that study, ranibizumab (intravitreal doses up to 2-mg) was repeatedly injected at 2- or 4-week intervals for 16 weeks in eyes with neovascular AMD. Surprisingly, 83% of eyes exhibited iridocyclitis although no serum anti-ranibizumab antibodies were detected.^24^ Recently, 52-week results of the CEDAR and SEQUOIA phase 3 clinical trials which compared the efficacy and safety of abicipar with those of ranibizumab in neovascular AMD were published. Abicipar is a pegylated designed ankyrin repeat protein that binds all isoforms of VEGF-A with high affinity and specificity. Like brolucizumab, abicipar is designed as a strong and long-acting anti-VEGF agent. Abicipar binds to human VEGF-A165 with 90-fold higher affinity and has a longer intraocular half-life as compared to ranibizumab.^25^ The safety outcomes of CEDAR/SEQUOIA trials showed that the incidence of IOI was 15.4% in the abicipar Q8 group, 20.4% in the abicipar Q12 group, and 0.3% in the ranibizumab Q4 group.^25^ Although the immunogenicity of abicipar should be considered, the results might somewhat endorse our speculation that potent and continuous VEGF inhibition alone can cause IOI.

The mechanism of brolucizumab-associated IOI surely affects the treatment strategy. Among ocular adverse events after brolucizumab IVI, retinal artery occlusion is the most important one as it can cause irreversible loss of visual acuity and/or visual field. Recently proposed expert opinions reflect this and recommend to sift to strong and intensive treatment in cases of retinal vascular occlusions combined with IOI.^8^^,^^10^ The currently recommended treatment strategy is local and systemic corticosteroid administration to reduce IOI, and we completely agree to this strategy. However, considering the above-mentioned impaired vascular endothelial scenario, what we treated with corticosteroid might be the secondary inflammation. While acute inflammation occurred in eyes with uveitis such as Behçet's disease and sarcoidosis can subside within 1 weeks after sub-Tenon's injection of triamcinolone acetonide, previously reported cases took more time to diminish inflammation.^3^^,^^14^^,^^16^ We speculate this could be explained as follows. Immune reaction to brolucizumab in the vitreous space decreased but continued as far as disrupted inner blood retinal barrier (iBRB) allows immune cells to migrate towards targets (brolucizumab). Then, with lapse of time, dose of intraocular brolucizumab decreased, and retinal vascular endothelial cells recovered their physiological functions including iBRB. After iBRB was restored, immune reaction to brolucizumab dramatically declined (please be notified that iBRB is one of the pivotal components responsible for ocular immune privilege^26^). If this speculation is justified, we have no choice but to wait and see for the management of brolucizumab-induced vascular damage. Therefore, the point might be to distinguish eyes with overt risks factors for vascular impairment from those without and to avoid brolucizumab IVI in eyes with apparent risk factors.

## 4 Conclusion

We have reported here a case of delayed-onset retinal artery occlusions and intraocular inflammation (IOI) following brolucizumab IVI. Because the onset of retinal vascular occlusion and IOI was several hours after we confirmed that no apparent abnormal findings exist on FA/IA, the event did not stem from the exacerbation of subclinical inflammation. Together with the fact that FA/IA performed at active phase of IOI did not show findings responsible for retinal artery occlusions, immune reaction to the drug alone could not explain what happened in this case. Although currently it is just a speculation, potent and prolonged VEGF inhibition by brolucizumab IVI might have caused severe damage to retinal vascular endothelial cells which subsequently led to (1) retinal vascular occlusions and (2) enhanced immune reaction to brolucizumab through the migration of immune cells towards vitreous cavity being allowed by the disrupted endothelial barrier function.

## Patient consent

Consent to publish the case report was obtained. Moreover, this report does not contain any personal information that could lead to the identification of the patient. This study complied with the tenets of the Declaration of Helsinki. Approval by the IRB was exempted as this is a single case report.

## Funding

No funding or grand support was received for this report.

## Authorship

All authors attest that they meet the current ICMJE criteria for authorship.

## Declaration of competing interest

None of the authors have financial disclosures or conflicts of interest relating to this topic.
